# The role of clinical associates in South Africa as a health workforce: A scoping review

**DOI:** 10.4102/phcfm.v16i1.4421

**Published:** 2024-05-09

**Authors:** Sanele Ngcobo, Lynn Bust, Ian Couper, Kathryn Chu

**Affiliations:** 1 Department of Family Medicine, Faculty of Health Sciences, University of Pretoria, Pretoria, South Africa; 2 Centre for Global Surgery, Department of Global Health, Faculty of Medicine, Stellenbosch University, Stellenbosch, South Africa; 3 Department of Global Health, Faculty of Medicine, Stellenbosch University, Stellenbosch, South Africa

**Keywords:** clinical associates, human resources for health, bachelor of clinical medical practice, district hospitals, clinical officers, physician assistants, physician associates, mid-level health workers, mid-level medical workers

## Abstract

**Background:**

South Africa’s health care system grapples with persistent challenges, including health care provider shortages and disparities in distribution. In response, the government introduced clinical associates (Clin-As) as a novel category of health care providers.

**Aim:**

This study mapped Clin-As’ history and practice in South Africa, assessing their roles in the health workforce and offering recommendations.

**Methods:**

Following the framework outlined by Arksey and O’Malley, we conducted a comprehensive literature search from January 2001 to November 2021, utilising PubMed, Scopus and EBSCOhost databases. One thousand six hundred and seventy-two articles were identified and then refined to 36 through title, abstract and full-text screening.

**Results:**

Strengths of the Clin-A cadre included addressing rural workforce shortages and offering cost-effective health care in rural areas. Challenges to the success of the cadre included stakeholder resistance, rapid implementation, scope of practice ambiguity, inadequate supervision, unclear roles, limited Department of Health (NDoH) support, funding deficits, Clin-As’ perceived underpayment and overwork, degree recognition issues, inadequate medical student training on Clin-A roles, vague career paths and uneven provincial participation.

**Conclusion:**

As a health care provider cadre, Clin-As have been welcomed by multiple stakeholders and could potentially be a valuable resource for South Africa’s health care system, but they face substantial challenges. Realising their full potential necessitates enhanced engagement, improved implementation strategies and precise scope definition.

**Contribution:**

This study acknowledges Clin-As in SA as a promising solution to health care workforce shortages but highlights challenges such as stakeholder resistance, insufficient NDoH support and unclear policies, emphasising the need for comprehensive efforts to maximise their potential.

## Introduction

Universal health coverage (UHC) is an essential human right. An important component of UHC is appropriate human resources for health (HRH) distributed to match population needs. In South Africa, there is a dire lack of HRH in rural areas. A key strategy to address the HRH crisis is task-sharing, or the use of mid-level medical workers (MLMWs) to perform certain tasks previously performed by doctors. Mid-level medical workers are endorsed by the World Health Organisation^1^ and are known as physician assistants, physician associates, clinical officers and others. In 2010, 47 of 54 African countries recognised and utilised MLMWs in various roles such as primary care, non-communicable disease, human immunodeficiency virus (HIV) tuberculosis and surgery.^2,3^ Mid-level medical workers’ clinical outcomes have been shown to be equivalent to those of doctors in certain clinical areas, provided that they receive adequate training, support and supervision.^4,5,6^

In rural South Africa (SA), district hospitals (DHs) are the backbone of the public sector hospital system but have historically been neglected and are chronically understaffed and underfunded. One of the government’s responses to these gaps has been the creation of a new category of health care provider in South Africa, clinical associates (Clin-As). Clinical associates are MLMWs intended to be employed in understaffed facilities in the public sector in SA to work towards achieving UHC and implementation of National Health Insurance (NHI) in SA.

Clin-A training programme was introduced at Walter Sisulu University in 2008, followed by the University of the Witwatersrand and the University of Pretoria in 2009, as part of a strategy to improve DHs. Clinical associates complete a 3-year undergraduate Bachelor of Clinical Medical Practice (BCMP). Clinical associates receive training and engage in practice across various health care settings, including community-based care, clinics, community health model centres (CHCs) and DHs. This comprehensive approach emphasises the importance of delivering health care services at multiple levels, ensuring that Clin-As are equipped to serve patients in diverse settings and manage common illnesses and diseases. As of December 2018, there were over 1000 Clin-A graduates in SA, with more than 80% working in the public sector in all nine provinces of the country.^7^ Regulations require Clin-As to work under the supervision of medical practitioners^8^; however, rural hospitals often have a shortage of doctors.

Although Clin-As have existed in SA for over 14 years, Clin-As in SA are poorly understood, and little has been written about their uptake in the health system. There is a gap in knowledge in terms of current evidence of Clin-A practice in SA. The HRH Effort Index serves as a valuable instrument for evaluating and guiding strategic investments in health care workforce development.^9^ It has seven HRH dimensions: Leadership and Advocacy; Policy and Governance; Finance; Education and Training; Recruitment, Distribution and Retention; Human Resources Management; and Monitoring, Evaluation and Information Systems.^9^ The HRH Effort Index, with its diverse dimensions, serves as a valuable tool for systematically reporting on a range of aspects of professional cadres in relation to the health workforce. Its integration within a scoping review offers a useful framework for organising the findings and identifying strengths, weaknesses and recommendations for future development of the cadre.

This study aimed to map the literature on Clin-As in South Africa, describe their history and practice, and assess their current roles using the HRH Effort Index.

## Methods

### Study design

This scoping review followed the methodological framework outlined by Arksey and O’Malley.^10^ A scoping review was chosen rather than a systematic review given the intention to map existing literature about Clin-As in SA since their initiation and provide a better understanding of their roles and contributions to the health workforce of the country. The scoping review was conducted by (1) identifying a clear research objective and search strategy, (2) identifying relevant research articles, (3) selecting research articles, (4) extracting and charting data, and (5) summarising, discussing, analysing and reporting the results. The HRH Effort Index was used as part of the final stage to support the analysis and organisation of the results.

### Literature search strategies

The literature search queried PubMed, Scopus and EBSCOhost (Academic Search Premier, Africa-Wide Information, CINAHL, Health Source: Nursing/Academic Edition, MEDLINE) databases from January 2001 to November 2021. The concept of utilising an MLMW in SA was re-introduced in 2001 and the idea existed from the 1940s but was never implemented; therefore, the search was started this year {Tollman, 1994 #37380}. Search terms included ‘clinical associate’ and variations thereof, and ‘South Africa’ (see Table 1).

**TABLE 1:** Search terms used.

Terms	Various terms searched
Clinical Associate	“clinical associates” OR “non-physician clinician” OR “non-physician clinicians” OR “non-physician practitioner” OR “non-physician practitioners” OR “mid-level provider” OR “mid-level providers” OR “mid-level health provider” OR “mid-level health providers” OR “mid-level healthcare provider” OR “mid-level healthcare providers” OR “mid-level health practitioner” OR “mid-level health practitioners” OR “mid-level healthcare practitioner” OR “mid-level healthcare practitioners” OR “mid-level practitioner” OR “mid-level practitioners” OR “mid-level health worker” OR “mid-level health workers” OR “mid-level healthcare worker” OR “mid-level healthcare workers” OR “clinical officer” OR “clinical officers” OR “substitute health worker” OR “substitute health workers” OR “advanced practice provider” OR “advanced practice providers” OR “assistant practice clinician” OR “assistant practice clinicians” OR “physician assistant” OR “physician assistants”
South Africa	

### Identification and selection of relevant studies

**FIGURE 1:**
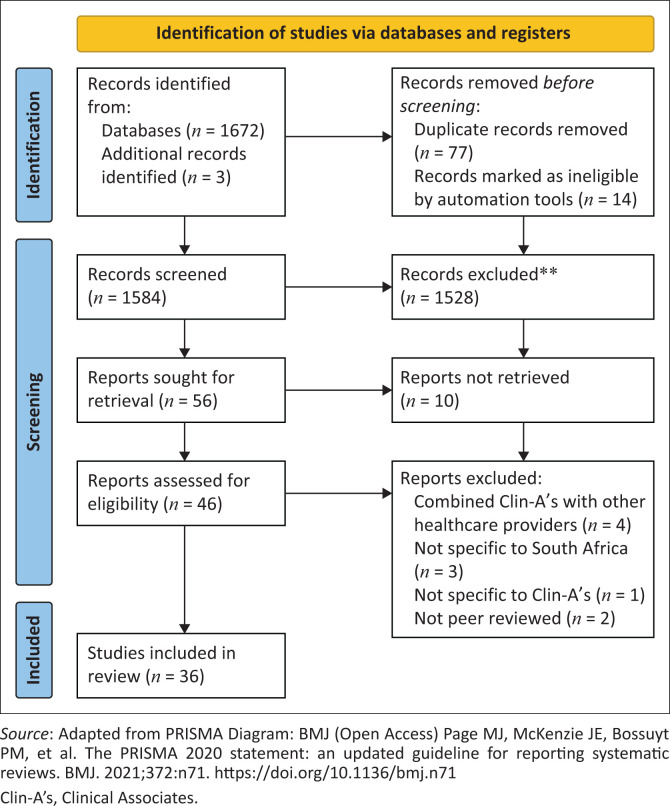
Preferred Reporting Items for Systematic Reviews and Meta-Analyses (PRISMA) flow diagram for the scoping review process.

The search produced 1672 articles. Title and abstract screening (by L.B. and V.Q.) narrowed the field to 56. The inclusion criteria encompassed studies published on Clin-As in South Africa in English language journals from 2001 to 2021. Both primary studies and opinion pieces, including narrative reviews and commentaries, were considered. Exclusion criteria involved non-journal publications, grey literature, studies without clear disaggregation of Clin-As from other health care providers or the South African context from that of other countries, and those without full-text availability. L.B. and S.N. independently reviewed the full text of the selected articles and, in the end, 36 articles were included. Figure 1 displays a flow diagram, adhering to the Preferred Reporting Items for Systematic Reviews and Meta-Analyses (PRISMA) guidelines that illustrates the procedure for searching and choosing research articles.

### Data extraction from the included studies

Data were extracted onto a prepared spreadsheet in Excel which included aim, study design, study setting, study population, overall focus of study, findings and future recommendations. Each extraction was done by two authors (L.B. and S.N.). In cases of discordance, L.B. and S.N. reviewed the article one more time, and if no agreement could be reached, K.C. was consulted to make the final decision. A descriptive analysis of all included literature was then undertaken, organised by the identified focus of each study. Contingent with scoping review guidelines, a quality appraisal of included articles was not conducted.

### Ethical considerations

This article followed all ethical standards for research without direct contact with human or animal subjects.

## Review findings

### Scoping review

The search resulted in a total of 1584 abstracts after the removal of duplicates. Thirty-six articles met the inclusion criteria (Figure 1). Just over half (*n* = 20; 56%) of the studies were empirical. Most of those (*n* = 17) employed observational or non-interventional methods, including 14 descriptive studies (seven surveys, two interview studies, one case study and four multi-method studies), and three comparative studies (one retrospective and two prospective studies) (Table 2). Two studies were intervention studies (one randomised controlled case study and one single cohort pre-post design). The remaining empirical study classified as ‘other’ was a cost-effectiveness study. The non-empirical studies (*n* = 16; 44%) comprised four commentaries, three editorials, three literature reviews, two letters to the editor and four ‘other’ descriptive studies.

**TABLE 2:** Summary of included studies.

Authors	Year	Title	Study design
Couper^28^	2014	Physician assistants in South Africa	Commentary
Rispel et al.^27^	2019	Socio-economic characteristics and career intentions of the WiSDOM health professional cohort in South Africa	Comparative study: Prospective longitudinal cohort study
Moodley et al.^30^	2020	Trends in practice intentions and preferences of clinical associate students: Implications for training and health services in South Africa	Comparative study: Prospective longitudinal cohort study
Ngcobo et al.^6^	2018	The quality of voluntary medical male circumcision done by mid-level workers in Tshwane District, South Africa: A retrospective analysis	Cross-sectional
Mapukata-Sondzaba et al.^37^	2014	Developing personal attributes of professionalism during clinical rotations: views of final year bachelor of clinical medical practice students	Descriptive study: Case study
Hamm et al.^16^	2016	Cost-effectiveness of clinical associates: A case study for the Mpumalanga province in South Africa	Descriptive study: Case study
Bac et al.^14^	2017	A new health care profession in rural district hospitals: A case study of the introduction of Clinical Associates in Shongwe hospital	Descriptive study: Interviews and literature review
Moodley et al.^23^	2014	Practice intentions of clinical associate students at the University of Pretoria, South Africa	Descriptive study: Survey
Dreyer et al.^39^	2015	Clinical Associate students’ perception of the educational environment at the University of the Witwatersrand, Johannesburg	Descriptive study: Survey
Memon et al.^36^	2016	Students’ perceptions of the instructional quality of district hospital-based training	Descriptive study: Survey
Monicca et al.^22^	2016	The effectiveness of clinical associates in addressing the human resources challenge of skills shortage: A case study of Tshwane District Hospital (South Africa)	Descriptive study: Survey
Monareng et al.^33^	2019	Practice choices of clinical associates: Policy realisation or practical reality?	Descriptive study: Survey
Koortzen et al.^34^	2020	Final-year medical students need to know their future supervisory role of clinical associates	Descriptive study: Survey
Vincent-Lambert et al., 2021^53^	2021	Doctors’, nurses’ and clinical associates’ understanding of emergency care practitioners	Descriptive study: Survey
Isembatya et al.^15^	2022	Clinical associates and access to health care in the Eastern Cape province of South Africa	Descriptive study: Surveys and interviews
J. F. M. Hugo, 2004^8^	2004	Implementation plan for a midlevel medical worker for South Africa: A discussion paper	Discussion paper
J. Hugo, 2004^24^	2004	Mid-level medical worker: Problem or Solution?	Editorial
Van Niekerk^19^	2006	Mid-level workers: High-level bungling?	Editorial
Couper et al.^32^	2007	Mid-level workers: High-level bungling?	Editorial
Doherty et al.^29^	2012	Will clinical associates be effective for South Africa?	Editorial
Hugo et al.^42^	2012	The clinical associate curriculum – The learning theory underpinning the BCMP programme at the University of Pretoria	Editorial
Ballweg^25^	2017	Best practices in PA development: Lessons from three countries	Editorial
Kerlen & Ballweg^13^	2017	One PA’s experiences in the Netherlands, South Africa and Australia	Editorial
Ngcobo^17^	2019	Clinical associates in South Africa	Editorial
Rogers^43^	2016	Does a brief workshop change clinical associate students’ resilience?	Intervention study: Pre–post single cohort
Smalley^35^	2018	PAs in South Africa	Letter to the editor
Gilbert^41^	2013	‘Re-engineering the workforce to meet service needs’: Exploring ‘task-shifting’ in South Africa in the context of HIV/AIDS and antiretroviral therapy	Literature review
Doherty et al.^11^	2013	Developing a new mid-level health worker: Lessons from South Africa’s experience with clinical associates	Mixed method
Mofolo et al.^21^	2019	Towards national health insurance: Alignment of strategic human resources in South Africa	Narrative review
Tshabalala et al.^7^	2019	Clinical associates in South Africa: Optimising their contribution to the health system	Narrative review
Couper & Hugo^12^	2014	Addressing the shortage of health professionals in South Africa through the development of a new cadre of health worker: The creation of Clinical Associates	Project report
Couper et al.^26^	2018	Curriculum and training needs of mid-level health workers in Africa: A situational review from Kenya, Nigeria, South Africa and Uganda	Qualitative
Louw and Hugo^38^	2020	Learning person-centred consultation skills in clinical medicine: A randomised controlled case study	Qualitative
Smalley et al.^18^	2020	Opinions of supervisors of clinical associates in South African district hospitals	Qualitative
Louw et al.^44^	2021	A capability approach analysis of student perspectives of a medical consultation quality-improvement process	Qualitative: Focus group discussions and reports
Kakia and Couper^31^	2021	Preceptors’ perceptions of assessing clinical associate students at district hospital sites	Qualitative: Interviews

*Source:* Adapted from PRISMA Diagram: BMJ (Open Access) Page MJ, McKenzie JE, Bossuyt PM, et al. The PRISMA 2020 statement: an updated guideline for reporting systematic reviews. BMJ. 2021;372:n71. https://doi.org/10.1136/bmj.n71

Note: Please see the full reference list of the article,Ngcobo S, Bust L, Couper I, Chu K. The role of clinical associates in South Africa as a health workforce: A scoping review. Afr J Prm Health Care Fam Med. 2024;16(1), a4421. https://doi.org/10.4102/phcfm.v16i1.4421, for more information.

Findings were summarised using seven recognised HRH dimensions (Table 3): Policy and Governance; Leadership and Advocacy; Finance; Education and Training; Recruitment, Distribution and Retention; Human Resources Management; and Monitoring, Evaluation and Information Systems.^9^

**TABLE 3:** Strengths, challenges and recommendations for clinical associates implementation in South Africa.

Theme	Strengths	Challenges	Recommendations
Policy and governance	Consultation with a wide variety of stakeholders^11^ Developed to fill a recognised workforce shortage in rural DHs^12^ Savvy policymaking and training implementation process^11^ Competency-based scope of practice tailored to the specific context and need of DHs^13^ Work in different units in DHs^14,15^ Work under supervision of medical doctors in a team^14,16^ HPCSA regulated profession^7,17^	Rapid implementation overwhelmed administrative capacity^11^ Lack of clear scope of practice at start^18^ Delays in formalising the scope of practice Lack of adequate supervision^7,12,15,19,20^ Lack of clarity regarding roles relative to other health care providers^14,18,21,22^ Unable to prescribe medication without a co-signature^7,14,17,18^ Degree not recognised outside of South Africa^23^ No intention of working independently^24^	Adapt Clin-As concept to country-specific health system challenges^25^ A higher level of political commitment to support Clin-As programme in South Africa is needed^26^ Clear policy direction on Clin-As is needed^27^ Review the scope of practice^7^ Incorporate Clin-As in an HRH strategy^7^
Leadership and advocacy	Clin-As self-organised and formed the Professional Association of Clin-As in South Africa^7,28^ Concept championed by three local universities^12^ Twinning of American-based Physician Assistant and South African-based Clin-As training programme supported by AIHA^12,25^ Support from RUDASA and family doctors also played a role in initial successes^13^	NDoH’s support for Clin-As has been limited^17^ Inadequate planning by provincial departments^12^ Lack of sustained political will11 Certain key stakeholders were against the concept^19^ Certain stakeholders felt there was no adequate consultation^19^	More effort should be put on advertising and advocating for this profession^17^ The role of a professional association must be strengthened^7^ DoH leadership to make the public sector the employer of choice^29^
Finance	Clin-As are a cost-effective strategy to meet the need for rural health care providers^16,24^ Clin-As are efficient (increase outputs and reduce costs) and effective (increase productivity, and quantity and quality of care).^7^	Lack of funding of posts to accommodate new graduates Funding shortfall^7,30^ Insufficient funding for training programmes^26^ Clin-As underpaid and overworked^17^	Establishment of a sustainable funding sources for training and deployment of Clin-As^20^
Education and training	Clin-As clinical training mostly takes part at family medicine training facilities led by Family Doctors.^24^ Practical training with integrated, case-based learning and early clinical involvement.^17,31^ Emphasis on generalist skills with flexibility in response to local needs.^21,29,32^ Training mostly in DHs in rural areas where the biggest need is.^14,33^ The education strategy is patient-centred and self-directed with an emphasis on self-learning.^22,34^ The national curriculum framework guides participating universities and ensures a common standard while allowing local differences and protecting university autonomy.^29^ Common exam for all three universities to ensure same minimum standard of competency.^13^ Emergency medicine postgraduate degree introduced at one university, further specialisations being explored.^35^ Perceptions of instructional quality of DH-based high.^36^ High pass rates of for the first group of cohorts.^29^	Lack of uptake of Clin-A programme by some medical schools^17^ First cohorts of Clin-A felt like guinea pigs because of the evolving curriculum^37^ Assessment process not clear to some preceptors^31^ Gap in medical student training to work directly with Clin-As become familiar with their scope of practice^34^ Medical students felt inadequately trained to supervise Clin-As in practice^34^ Insufficient focus and knowledge of basic science^15^	Scale up production of Cln-As^7,11,20^ Update training method, with a specific focus on problem-solving approaches, and include more life-saving procedures^26^ Align BCMP curriculum with the burden of diseases^26^ Train other health care providers on the scope of practice and role of Clin-As^34^ Strengthen interprofessional education in medical schools^34^
Service delivery Recruitment, distribution, and retention	Clin-As provide the same quality of care as high-level workers provided they receive adequate training, support, and supervision.^6,16^ Clin-As able to take responsibility for some wards and departments with supervision.^15^ Improve workload for doctors.^6,15,18^ Provide opportunities to people from marginalised communities.^17^ No language barriers.^29^ The majority of Clin-As intended to practice in rural areas throughout SA.^23^ Intention to practice in a rural area was associated with living in a rural area, and rural DH exposure during training.^23^ Expands government DH health staff.^18,20^	Almost half of Clin-As intended a career change^33^ Lack of clear career paths.^17^ Shortfall of Clin-A production below national targets^7^ Risk of exploitation in the private sector^29^	Clin-As must also work in CHCs and clinics^14^ Further research should be done to better understand the reasons for an increase in intentions to change careers^33^
Human resource management	Solution to human resource constraints in DHs.^20,22^ Reduction of human resource challenges of skills in DHs^22^ Improved task sharing^22^ Health care providers working with Clin-As recognised them as important parts of the team, with positive encounters.^15,18,22,34^	Not all SA provinces are participating; Western Cape refuses to employ ClinAs.^12^ Insufficient posts in the public sector^7,11^ No recruitment strategies^22^ Lack of career paths^21^	Develop strategies to manage tensions between different categories of health care providers^11^ Conduct Clin-A job reevaluation in the public sector^7^
Monitoring, evaluation, and information systems	Quality improvement intervention for students a valuable opportunity to reflect and gain person-centred skills and competencies.^38^	Output of trained Clin-As does not meet the estimated target^13^	Periodical evaluation of Clin-As students’ perceptions of the BCMP learning environment is needed^36,39^ Constantly share BCMP-related research results with all stakeholders involved in training BCMP students^36^ Assess the initial impact of the new cadre on health services^20^

*Source:* Adapted from PRISMA Diagram: BMJ (Open Access) Page MJ, McKenzie JE, Bossuyt PM, et al. The PRISMA 2020 statement: an updated guideline for reporting systematic reviews. BMJ. 2021;372:n71. https://doi.org/10.1136/bmj.n71

DH, district hospitals; HPCSA, Health Professions Council of South Africa; AIHA, American International Health Alliance; RUDASA, Rural Doctors Association of South Africa; DoH, Department of Health; BCMP, Bachelor of Clinical Medical Practice; CHCs, Community health centres; Clin-As, clinical associates; HRH, human resources for health.

Note: Please see the full reference list of the article,Ngcobo S, Bust L, Couper I, Chu K. The role of clinical associates in South Africa as a health workforce: A scoping review. Afr J Prm Health Care Fam Med. 2024;16(1), a4421. https://doi.org/10.4102/phcfm.v16i1.4421, for more information.

#### Policy and governance

The concept of an MLMW cadre in SA originated in the 2001 Pick Report.^8^ In 2003, the Minister of Health made a decision, confirmed in an executive meeting in 2004, that an MLMW programme would be implemented in SA.^8^ Positions similar to MLMWs existed in both colonial and post-colonial SA, such as hospital orderlies and medical assistants.^40^ Their historical design and functions were to fit into a less prominent status within the prevailing racial and professional hierarchies, which were predominantly under the control of white personnel.^40^ At the start of the implementation of the Clin-A programme, there was some concern that Clin-As could have an unsatisfactory relationship with doctors and nurses because of unclear boundary roles, and potential challenges of power and control with task-shifting.^29,41^ Therefore, the term *Clinical Associate* was chosen to avoid any sense of subservience in a newly democratic state. ‘Associate’ was considered more egalitarian than ‘assistant’. In order to shift from the biomedical to a holistic model, the term ‘clinical’ was chosen over ‘medical’.^28^

The vision for MLMWs was that they would work in synergy with primary health care teams, under the supervision of medical doctors.^8^ With less cost and time in training, the aim was to relieve the human resources crisis and fill gaps in workforce shortages in public DHs, especially in rural areas where doctor posts often remained unfilled.^14,41^ The programme would increase the number and quality of health care providers at the district level, improve health care access for marginalised communities and reduce the need for referrals from district to higher-level services.^21,29^ Digby et al. reported that the Clin-As were designed to bridge the divide between urban and rural areas as well as between well-resourced and underserved regions of the country, with no intention to replace any existing cadre of qualified health professionals.^40^ The intended benefits of Clin-As included their ability to perform tasks with generalist skills and flexibility which would enable other health care providers to focus on their own roles to provide better quality care.^32^

In addition, students were purposively selected from rural areas into the Clin-A programme in order to provide training opportunities to marginalised communities, reducing the likelihood of brain drain.^29^ An early target was set to produce 1350 Clin-As, based on the intention to reach five Clin-As per DH, nationally.^29^

A task team was appointed and recommended that an approach be developed aimed at starting with training and roll-out of the programme at district-level services. Early on, different key players in developing a Clin-A programme were identified including provincial and national Departments of Health (DoH), universities and medical schools, the Health Professions Council of South Africa (HPCSA), other health workers in DHs, and the Departments of National Education and Labour.^8^ There was extensive consultation at the start of implementation, including workshops and engagement with a broad range of stakeholders.^13,32^ Doherty et al. reiterated that stakeholder interests and sensitivities were taken into account at the introduction of the programme, and commended the initial process for its savvy policy-making and training implementation processes.^11^ Successful uptake of the Clin-A training programmes at three SA universities was credited to the presence of local champions.^12,13^ Buy-in from the Rural Doctors Association of South Africa (RUDASA) and family doctors also contributed to initial successes.^13^

The profession was established with the initial support of the American International Health Alliance (AIHA) twinning project which linked each Clin-A training programme with a United States training physician assistant (equivalent to Clin-As) faculty.^25^ Clin-A graduates organised themselves at an early stage to form the Professional Association of Clin-As in September 2012, after being inspired by attending the American Academy of Physician Assistants conference and student conference.^12^

#### Scope of practice

Clinical associates were developed as MLMWs, trained to do various primary care tasks that would partially relieve the clinical duties of doctors and nurses. It was noted their scope of practice was intended to be competency-based, and specifically tailored to the context and needs of DHs.^29^ Under the supervision of medical doctors,^8^ they would work in different units of government DHs including emergency, maternity, outpatient, surgical, medical and paediatric departments. Initially, mechanisms were not in place for Clin-As to be employed in the private sector.^28^ When the profession was introduced, there was no formalised scope of practice or job description; however, the scope of practice for Clin-As was signed by the national Minister of Health in November 2016.^17,18^

Clinical associates are expected to offer holistic care to patients with a range of common conditions and have consultation, physical examination and counselling skills.^28,29^ Competency development was based on the most common conditions seen, and procedures and skills performed in DHs.^12^ Tasks include assisting with the provision of emergency care, conducting ward rounds, performing diagnostic and therapeutic procedures, and in-patient care.^29^

Regulation of Clin-As via the Medical and Dental Board of the HPCSA ensures maximum synergy with the medical profession.^8^ Because the role and regulation of Clin-As was not finalised until a later stage, their development and wider deployment were delayed.^21^ In addition, the poorly defined scope of practice for Clin-As allowed contestation between different health care providers and Clin-As.^14,40^ Digby et al. stated that ‘the ill-defined professional contours of the ‘middle’ could be enabling in the short term’ since posts were crucial for addressing employment gaps, although it could be destructive in the longer term as other professionals saw intermediaries as a threat to their own status.^40^

Several challenges around the scope of practice were reported by Cin-As. The lack of a well-defined career path or progression was a major reason that some reported the desire to leave their profession.^17^ Further concerns reported by Doherty et al. regarding their role included that they become a second-best option for poor and rural communities, there is a lack of appropriate supervision because of doctor shortages and there is potential for Clin-As to be exploited in the private sector.^29^

There was also contestation over the independence of Clin-As’ practice. The juxtaposition of Clin-As as ‘mid-level’ providers who assume responsibilities but under supervision was interpreted differently in different contexts.^40^ For example, the ability to prescribe medications is an essential need to complete clinical tasks.^29^ However, in 2013, Clin-As were prohibited from prescribing medications (without a doctor’s counter signature) by the Pharmacy Council, until new regulations were promulgated.^11^ During the introduction of Clin-As to Shongwe hospital in Mpumalanga, pharmacists refused to issue any medication prescribed by a Clin-A.^14^ In 2019, Clin-As were still not yet regulated as authorised prescribers which hampered their clinical practice.^7^

### Leadership and advocacy

National leadership and political support were noted to dwindle as the process unfolded, and consensus eroded as unexpected problems occurred.^29^ While there was strong support for Clin-As from some stakeholders, others felt there was a lack of adequate consultation during the development process.^19,25^ In an opinion piece by Van Niekerk, he suggested that the introduction of Clin-As might lead to professional boundary disputes, and implementation of the programme was against advice from key health worker organisations.^19^ Doherty et al. stated there was a lack of ongoing commitment from the national DoH, and a lack of buy-in from certain provinces such as the Western Cape, and universities.^11^ Two national task teams were commissioned to identify the challenges of the Clin-A programme: one did not complete the work and the other made a report with recommendations that have not been adopted by the DoH.^17^ These recommendations focused on the career path, review of the scope of practice, creation of posts, reevaluation of the Clin-As profession and improved conditions of service.

### Education and training

The training curriculum for Clin-As is a 3-year BCMP programme.^12^ The aim is to educate Clin-As in work-based clinical practice, underpinned with a sound understanding of family medicine principles.^7^ There is a strong focus on teaching the skills and knowledge to provide DH-level services, particularly in rural areas where there is the greatest shortage of doctors.^8,12^

As of this review, 3 of the 10 medical schools in SA provided Clin-A degree programmes: Walter Sisulu University enrolled their first cohort of 23 students in 2008, followed by the University of Pretoria (55 students) and University of Witwatersrand (25 students) in 2009.^29^ Each year, between 70 and 140 Clin-As graduate, and as of 2018, the three universities have produced 1070 graduates.^7,17^

Originally, the national strategy was to provide Clin-As training programmes at all faculties of health sciences in SA^8,28^; however, there was hesitation by some universities to be involved.^17^ As of 2018, two further universities were considered Clin-A programmes (Nelson Mandela Metropolitan University has received approval to start a programme, and the University of Venda in Limpopo has expressed interest), but they have yet to initiate programmes.^35^ Universities that partake in training were described as enthusiastic and committed, driven by local champions at the university.^12,29^

Training is standardised through a uniform curriculum but allows participating universities some flexibility in their curriculum development.^11^ The HPCSA has developed core competencies, and the undergraduate curricula incorporate six ‘Entrustable Professional Activities’ used as learning objectives to measure students’ competency including: perform patient assessment, manage patients comprehensively, promote health, facilitate communication and collaboration, improve health care services and develop professionally.^7^ There is a common exit exam (jointly formulated by all three universities) for the three universities to ensure a minimum standard of competency, which is unique in professions governed by the HPCSA.^7^ There was some initial contestation over the content of the curriculum, with some stakeholders believing there was too much content to fit into three years.^19,32^

Clinical training mainly takes place at the training hospitals linked to the three institutions in Eastern Cape, Gauteng, North West and Mpumalanga provinces.^12^ Students receive lectures on university campuses before spending most of their time in selected DHs.^11^ Training at the district level occurs at sites known as clinical learning centres with small groups (up to 12 students per year per training unit) focused on integrated learning with maximum exposure to real patients.^8,11^ Training is practically based; for example, a 3rd-year Clin-A is expected to work 972 normal hours and 272 after-hours a year.^17^

In 2013, Doherty et al. included interviews in which participants reported that initial training was of good quality with pass rates of approximately 95% for the first group of cohorts.^11^ The majority of students (between 65% and 90% from 2011 to 2017) were funded by bursaries, provided primarily by the provincial government and the military, rather than the national government.^12,30^ Couper et al. warned that a Clin-A career should not be a ‘dead end’, but there should rather be opportunities to gain specialist skills through in-service training or post-graduate courses.^12^ In 2018, to develop the academic pathway for Clin-As, a 1-year postgraduate degree in emergency medicine was offered by the University of Witwatersrand, with specialisations in obstetrics and anaesthesia also being explored.^30,35^

The training programme utilises a transformative educational model focused on self-regulated learning and includes authentic learning in multiple cycles, with emphasis on clinical training and action learning.^28^ The curriculum emphasises teamwork, with some shared clinical rotations with medical students.^12^ However, a survey of medical students at the University of Pretoria reported that there were no formalised opportunities in the curriculum for medical students to work in teams with Clin-As and become familiar with their scope of practice.^34^ During initial rotations at the programme’s inception, there were misunderstandings between Clin-A students, and doctors and nurses around the role of Clin-As and their learning objectives.^14^ However, this improved over time with increased student satisfaction reported at one clinical learning centre in Mpumalanga.^14^

Training has mostly been facilitated by small multidisciplinary teams of two to three staff, some of whom have joint appointments in academia and government health departments.^42^ Trainers, primarily stationed in DHs, are often essential members of the university’s family medicine training complex, demonstrating their crucial role in both clinical and educational settings.^8,11^ One study by Kakia and Couper reported that their trainers were enthusiastic, and supervised students in the clinical environment for practical experience.^31^ However, a shortage of Clin-As student trainers was reported and it was because of inadequate funding for their positions.^25^ One study assessed trainers’ perceptions of Clin-A assessments at DH sites, and found that there was a lack of training and support from universities for trainers, and identified conflict between students and trainers based on expectations of the assessment.^31^

Three studies investigated Clin-A’s perceptions of their learning environment.^36,37,39^ Findings indicated that the educational environment was perceived to prepare Clin-A students to be reflective practitioners and that the instructional quality was consistently seen to be high at clinical learning centres, particularly those that were more rural.^36,37^ Students became self-directed learners throughout the course of their studies, and trainer activities, learning opportunities and the learning environment were perceived to be of high quality in the second and third years of Clin-A studies.^36^ Students considered positive aspects of the academic climate to include student competence and confidence development, student participation in class, constructive criticism, empathy in practising medicine and friendships.^39^

However, as the programme was new, some students complained of feeling like ‘guinea pigs’, and felt vulnerable when pushed beyond their scope of work.^37^ Certain instances were reported when student learning and patient care were compromised; these were attributed to inadequate communication, lack of resources, and attitudes or shortages of health care providers.^37^ Areas of the educational environment recommended for improvement included feedback provision to students, course timetables, ensuring a non-stressful course, provision of good support systems and social life improvement.^39^

Three studies analysed interventions introduced in the educational environment.^38,43,44^ One educational intervention consisted of quality improvement training to improve person-centred practice of Clin-As.^38^ The process was reported by Clin-A students to be a valuable opportunity to self-evaluate and identify practice areas needing improvement to gain the required competencies.^44^ A further study measured change in person-centred practice over time as a result of the quality improvement intervention and found that there was not a significant effect when comparing the intervention to a control group.^38^ However, person-centred practice did improve over the study period suggesting improvements in consultation skills were gained in other ways throughout Clin-A training.^38^ Another study investigated whether a brief intervention changed Clin-A students’ resilience and reported high baseline resilience but found no significant changes after the workshop.^43^

### Finance

Supervisors of Clin-As in DHs reported that Clin-As were efficient (increased outputs and reduced costs) and effective (increased productivity, and quantity and quality of care) in practice.^7,18^ One case study found that training and employing Clin-As was potentially cost-effective, with Clin-As being able to free up the time of doctors. Furthermore, provided they receive adequate training, support and supervision, Clin-As can provide quality care.^16^ Despite some initial challenges with the introduction of Clin-As at one DH, it was found there was improved waiting times for patients.^14^ Doctors in this DH suggested that vacant medical officer posts could be converted to employ Clin-As to better serve patients without increasing the cost of human resources.^14^

Further challenges of the Clin-A programme included its rapid implementation which overwhelmed administrative capacity, and a funding shortfall of the programme because of a miscommunication between the DoH and Treasury.^21^ Delayed employment because of insufficient posts in the public sector, coupled with the potential of poor working conditions for Clin-As, were challenges to the career progression of the first cohort of graduates.^11^ Some Clin-As reported that the pay gap between their cadre and medical doctors was demoralising given their perceived value to the latter group.^11,14^

### Recruitment, distribution and retention

When the Clin-As programme was introduced, they were specifically recruited from rural areas, with three quarters of Clin-As coming from the lowest two socio-economic status quintiles.^27^ Clinical associates are more likely to be able to address patients without language barriers and be more easily retained in rural areas.^29^ With this ‘in-between’ set of skills and practice, Clin-As have been described as ‘culture brokers’ where they are able to bridge gaps between the sometimes foreign Western world of medicine and their patients.^40^

Four studies looked at the practice choices of Clin-As in SA.^16,23,30,33^ The proportion of Clin-A students intending to practice in rural areas ranged from 60% to 70% between 2011 and 2017, with intention to practice being significantly associated with having lived most of one’s life in a rural area, and rural DH exposure during training.^23,30^ The majority of Clin-As indicated a preference for the public sector and DHs were found to be the most preferred setting in two studies in 2014 and 2017.^30^ In practice, it was found that more than 80% of Clin-As were employed in the public sector across all nine provinces in SA, with 50% employed in rural areas.^7,33^ Factors such as where Clin-As spent most of their lives and bursary obligations influenced their practice choices.^33^

After completion of their studies, 90% of Clin-As from the University of Pretoria intended to partake in further studies, with 44%–67% reporting an intended career change.^23,27,33^ The risk that Clin-As will leave their profession is attributed to the lack of a clearly defined scope of clinical practice and career path, resulting in Clin-As being overworked and underpaid.^7,17^ In addition, only 14% of Clin-As said the profession was their first career choice which could contribute to the low retention rate.^27^

Clin-As have been employed in the government health sector from 2011.^29^ A qualitative study by Scot et al. reported Clin-As to be professional and able to assist doctors with good displays of clinical work.^18^ They have been reported to be able to take responsibility for some wards and departments with adequate supervision from doctors.^14^ Between 2010 and 2016, 794 Clin-As completed training, of which 720 commenced practice in SA.^21^ This is below the minimum target of 1350 Clin-As, and well below the National Task Team’s estimate of 11 500 required by 2030.^7^

One study examined outcomes of voluntary medical male circumcisions (VMMCs) carried out by Clin-As, and found that the VMMCs performed by Clin-As showed no significant differences in adverse events and operation times compared to those done by doctors.^6^ In the Eastern Cape, a survey conducted in 2013 reported that Clin-As were able to provide care to approximately 17 people living with HIV per day in the Eastern Cape.^7^ Clinical associates were shown to be effective as part of a multi-disciplinary team where together with social workers and community health workers, they worked in a Community Oriented Substance Use Programme in one district in SA.^7^

Despite some Clin-As expressing a desire to work overseas or open their own private practice, the degree is not internationally recognised and Clin-As are not permitted to have their own private practice in SA.^30^ Further challenges are a reported lack of independence as Clin-As require supervision for surgical procedures, and are unable to write prescriptions without co-signatures.^18^

### Human resource management

Challenges related to the practice of Clin-As in SA include a lack of funding to create posts to accommodate new graduates, and a lack of career paths to prevent potential brain drain.^21^ In addition, there has been reported growth of Clin-As in the private sector which has been linked to the failure of the provinces to continue student bursaries and create enough public sector posts.^7^

One study at a single DH found the majority of health care providers (doctors, nurses and allied health professionals) recognised Clin-As as important members of the team, and had positive encounters with them.^22^ Another study reported both acceptance and appreciation for the introduction of Clin-As by health care providers.^18^ Ngcobo reported an opinion of a family medicine registrar working with Clin-As reported that:

Academic-wise, they have more practical ways of approaching a case, they stimulate and challenge me academically as they have a lot of information, and their level of practice is very close to that of a general practitioner. They are skilled in making a diagnosis and managing patients.^17^

Since the introduction of Clin-As, there has been some controversy over their position within the medical hierarchy, with noted potential for interprofessional boundary disputes and power struggles.^41^ In a study carried out in Tshwane DH, despite being able to recognise Clin-As, the majority of health care providers did not understand Clin-A’s role or scope of practice. This was attributed to the fact that there was no formalised scope of practice for Clin-As until 2016 and a lack of information regarding the identity and training of Clin-As.^18,21,22^ Nurses were found to work most closely with Clin-A,^22^ but it was reported that they might feel apprehensive as Clin-As could threaten to take away some of their functions, and might occupy a higher status.^41^ Despite having worked with Clin-As, medical students in their final year appeared largely unaware of their obligation to supervise Clin-As in practice.^34^ However, one study at a single DH found the relationship between Clin-As and other health care providers improved over time after the introduction of Clin-As, once understanding and appreciation of their role grew.^14^

### Monitoring, evaluation and information systems

Quality improvement interventions in Clin-A training offered valuable opportunities for developing person-centred skills and competencies.^38^ Periodical evaluation of Clin-A student perceptions was necessary, along with sharing relevant research results with stakeholders involved in Clin-A training. Additionally, it’s important to note that the output of Clin-As by universities was reported to have fallen short of the estimated target.^39^

## Discussion and recommendations

This review of 36 studies mapped and synthesised the available literature on Clin-As in SA. These studies encompass editorials and discussions on the introduction and effectiveness of Clin-As in SA. Additionally, various research types, including descriptive studies, surveys, qualitative research, comparative studies and narrative reviews, explore topics such as educational environments, practice intentions, cost-effectiveness, career choices and the overall impact of Clin-As in health care delivery. The research provides a comprehensive overview of the development and outcomes of mid-level medical workers, focusing on Clin-As in diverse contexts.

All seven HRH index dimensions informed the synthesised evidence, with the articles yielding information. While Policy and Governance, Leadership and Advocacy, Education and Training, and Service Delivery Recruitment, Distribution and Retention received the most emphasis, valuable insights were still derived from Monitoring, Evaluation and Information Systems; Human Resource Management; and Finance.

The success of health policy interventions is dependent on political will, availability of funding, administrative capacity and intergovernmental bargaining.^45^ Our findings suggest that during the inception and planning phase, there was national and sub-national political commitment.^8^ However, planning between the DoH and National Treasury was not coordinated resulting in a lack of allocation of funds for Clin-A posts. Although the DoH is the employer of Clin-As, their employment conditions are established by the Department of Public Service and Administration. Their salaries are contingent on funding from the national treasury.

While a continuous need for intergovernmental negotiations exists, our findings point to insufficient evidence for such collaborative efforts.

A study done at the University of Pretoria found that 90% of Clin-As wanted to pursue further studies.^33^ As SA transitions to NHI, there is a need to clearly define the role of Clin-As in speciality care. District clinical specialist teams that were part of an NHI pilot intervention failed to adequately improve maternal and child health at the primary health care level as intended, and this was linked to the lack of gynaecologists and paediatricians in these teams.^46^ This factor needs to be considered as ongoing dialogues happen around postgraduate training for Clin-As.

Clin-A specialisation has been controversial with some stating it would be contradictory to the very ethos of this MLMW profession and others believing it would improve career progression. Sadler et al.^47^ view the specialisation of PAs in USMLMW as a threat to the profession by restricting versatility and comprehensive care provision especially given their role in rural district hospitals where generalists are needed. This is supported by Dovlo et al. who warned that lack of career progression is restrictive and may create frustration and low motivation among MLMWs.^48^ In Malawi, Kenya, Tanzania and Mozambique, MLMWs specialise in different fields including anaesthesia, ophthalmology, surgery, women’s health and orthopaedics. In accordance with the needs of SA’s health system, Clin-As can adopt a generalist approach with extended training, similar to the philosophy of family medicine. Another option is to embrace specialisation but limit it to clinic, CHCs and DH level, with focus in areas such as surgery, obstetrics, paediatrics and anaesthesia. This might be a solution to career progression for Clin-As while addressing shortages in specialised areas and reducing unnecessary referrals to higher levels of care. However, the notion of exclusive tertiary-level specialisation may not align optimally with the comprehensive health care demands within the South African context.

The profession of Clin-As in SA is categorised as a supervised practice. This is similar in high-income countries such as the US and UK. In contrast to this, many African lower-income countries have clinical officers functioning independently from medical practitioners, as seen in Malawi, Kenya, Zambia and others.^49^ Results from this review show that there are instances of lack of appropriate supervision of Clin-As in SA because of doctor shortages, but some studies did report adequate supervision. In a study carried out in the US, Physician Assistants practising in rural areas reported a broader scope of practice, more autonomy and less supervision by medical doctors than their peers in urban environments.^50^ Innovative supervision methods are crucial for Clin-As in SA because of limited doctor availability. Virtual supervision offers the opportunity to enhance supervision for Clin-As by offering remote guidance. Postgraduate qualifications in specialised areas might be a solution, offering an opportunity for advanced training and enhanced competence level resulting in full autonomy.

Much attention has been drawn to the independence and autonomy of PAs even in countries such as the US. A few years after the PA’s programme was introduced in the US, Schneller argued that professions that are built as subordinate to the more powerful and prestigious professions have built-in limits to the degree to which they can acquire valued professional attributes as they enter a ‘negotiated, semi-autonomous dependent relationship with the supervising doctor’.^51,52^

The majority of the literature on Clin-As identified in this scoping review consisted of opinion pieces and commentaries. Several areas within the scope of Clin-As’ responsibilities remain under-researched. These gaps include their role in community health, contributions to preventative medicine and involvement in community-oriented substance use programmes. Additionally, there are gaps in quantitative studies validating their impact, effectiveness within public and private health systems, and their involvement in the academic training of other Clin-As. These highlighted gaps are just examples among many other areas of research needing exploration, emphasising the broader need for a comprehensive investigation into the roles and impact of Clin-As in diverse health care domains. Addressing these research gaps is essential for a comprehensive understanding of the impact and potential of Clin-As across various domains.

## Conclusion

In conclusion, there is sparse literature on the implementation of Clin-As in SA. Few empirical studies exist and there are gaps in areas such as health system effectiveness and patient perceptions of their role. This review indicates that there are challenges with prescription rights, limited job opportunities, insufficient career advancement, supervision concerns and gaps in knowledge regarding Clin-As. By addressing these issues, South Africa can leverage Clin-As to significantly improve health care access, especially in underserved areas, and strengthen its health care system overall.
